# CT angiographic study of the cerebral deep veins around the vein of Galen

**DOI:** 10.7150/ijms.54891

**Published:** 2021-02-06

**Authors:** Kun Hou, Tiefeng Ji, Tengfei Luan, Jinlu Yu

**Affiliations:** 1 Department of Neurosurgery, The First Hospital of Jilin University, Changchun, 130021, China.; 2 Department of Radiology, The First Hospital of Jilin University, Changchun, 130021, China.

**Keywords:** basal vein, cerebral deep vein, computed tomography angiography, internal cerebral vein, straight sinus, vein of Galen

## Abstract

Research on the anatomy of cerebral deep veins (CDVs) around the vein of Galen (VG) is very important and has fundamental clinical significance. Large-scale anatomical studies of CDVs using computed tomography angiography (CTA) are rarely reported. A retrospective study of the CDVs around the VG was conducted in Chinese patients of Han nationality. One hundred cases were included in the final analysis. The patients were aged from 17 to 78 years (mean: 42.3 years). Also, 46% of the patients were female. The diameter of the internal cerebral vein (ICV) at its beginning and termination points ranged from 0.4 to 2.8 mm (1.49 ± 0.39 mm) and 0.4 to 3.5 mm (2.05 ± 0.47 mm), respectively. There was statistical significance regarding the diameter of the ICV at its beginning and termination points (P <0.01). The ICV length ranged from 28.5 to 47.9 mm (36.86 ± 3.74 mm). The length of the straight sinus (SS) ranged from 30.2 to 57.8 mm (43.6 ± 6.37 mm). The length of the VG ranged from 1.5 to 41.8 mm (9.30 ± 4.76 mm). The angle at the VG and SS transition area ranged from 25.4 to 110.6° (77.2 ± 18.0°). This study was a meaningful attempt to conduct anatomical research of CDVs using CTA. Preoperative familiarity with the normal venous structure and its variation around the VG would be helpful for endovascular treatment.

## Introduction

The internal cerebral vein (ICV) together with the basal vein of Rosenthal (BV) and their tributaries form the cerebral deep vein (CDV) system 1. Research on the anatomy of the deep veins around the vein of Galen (VG) is very important and has valuable clinical significance. In general, CDVs are highly variable, but the VG, ICV, and BV are relatively constant 2.

Most of the previous anatomical studies on CDVs were performed through autopsy or catheter angiography 3. Large-scale anatomical studies of CDVs using computed tomography angiography (CTA) are rarely reported. CTA is an easier, accessible and noninvasive method of investigation and supports powerful postprocessing, which signifies its important role in anatomical research. Recently, we performed an anatomical study of the superficial temporal artery using CTA on a GE workstation 4. The GE workstation has a variety of unique functions and tools in 3-dimensional reconstruction and measurement, which permits advanced study of the vascular anatomy. After 3-dimensional reconstruction, CTA can clearly display CDVs. Therefore, this study conducted a large-scale radiological anatomical study of the CDVs around the VG using CTA data.

## Materials and methods

**Figure 1:**
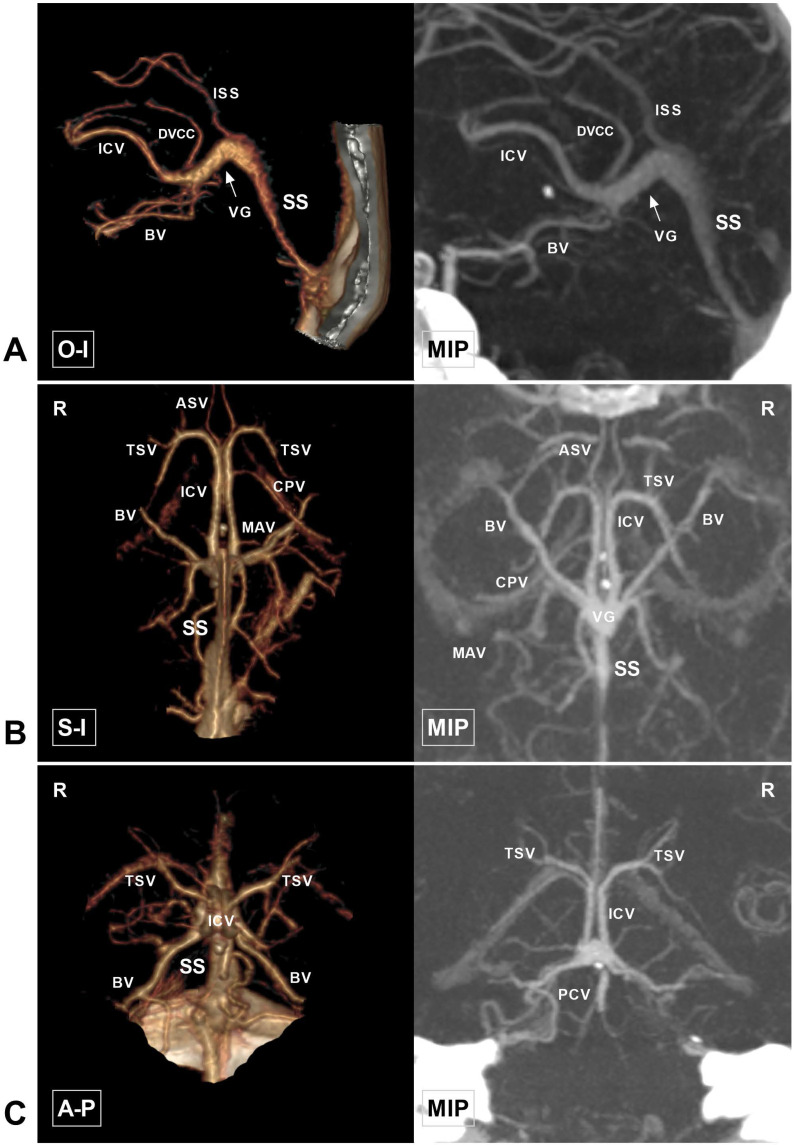
** Anatomy of the cerebral deep vein on CTA. A,** CTA and MIP in the O-I view show that the ICV extends backward as the VG (arrow). The VG continues as the SS and empties into the torcular herophili. The DVCC and ISS drain into the VG at their beginning and terminal portions, respectively. **B,** CTA and MIP in the S-I view show that TSV and ASV merge into the ICV. The bilateral ICVs and BVs merge into the VG and continue as the SS. The CPV and MAV draining into the ICV are also visible. **C,** CTA and MIP in the A-P view show that the TSV continues as the ICV and then merges with the BV. The PCV draining to the VG is visible. **Abbreviations:** A-P, anterior to posterior; ASV, anterior septal vein; BV, basal vein; CPV, choroid plexus vein; CTA, computed tomography angiography; DVCC, dorsal vein of the corpus callosum; ICV, internal cerebral vein; ISS, inferior sagittal sinus; MAV, medial atrial vein; MIP, maximum intensity projection; O-I, outside to inside; PCV, precentral cerebellar vein; R, right; S-I, superior to inferior; SS, straight sinus; TSV, thalamostriate vein; VG, vein of Galen.

A retrospective study was conducted with Chinese patients of Han nationality who underwent head CTA examination between April 2019 and April 2020 at our institution. A total of 100 consecutive patients who met the inclusion criteria were selected for further investigation. This study was approved by the Ethics Committee of The First Hospital of Jilin University (approval number: 2020-327). The original CTA data were further processed on a GE workstation (version 4.6; GE Healthcare). The general anatomy of CDVs around the VG in a normal patient is illustrated in Figure 1.

### Inclusion and exclusion criteria

1. Satisfactory contrast agent filling of the CVD system.

2. No intracranial space-occupying diseases or vascular diseases that might affect the morphology or blood flow to the CVDs around the VG, such as Galen aneurysm, cerebral arteriovenous malformations, dural arteriovenous fistula, and adjacent occupying lesion, were observed.

### Data processing and measuring

Raw CTA data were analyzed using a GE workstation. The original data were reconstructed using the Volume Rendering program. The nonvenous structures surrounding the VG were removed using the cut tool. The internal diameter of the vessels and the spatial distance between them were measured using the Measure distance tool. The Two Click AVA tool was used to measure the length of the vessels. The angle between different vessels was measured with the angle tool in Photoshop (2020, US. Adobe Systems Incorporated). Each parameter was measured three times, and the average value was used in analysis.

### Measurement parameters

**Figure 2:**
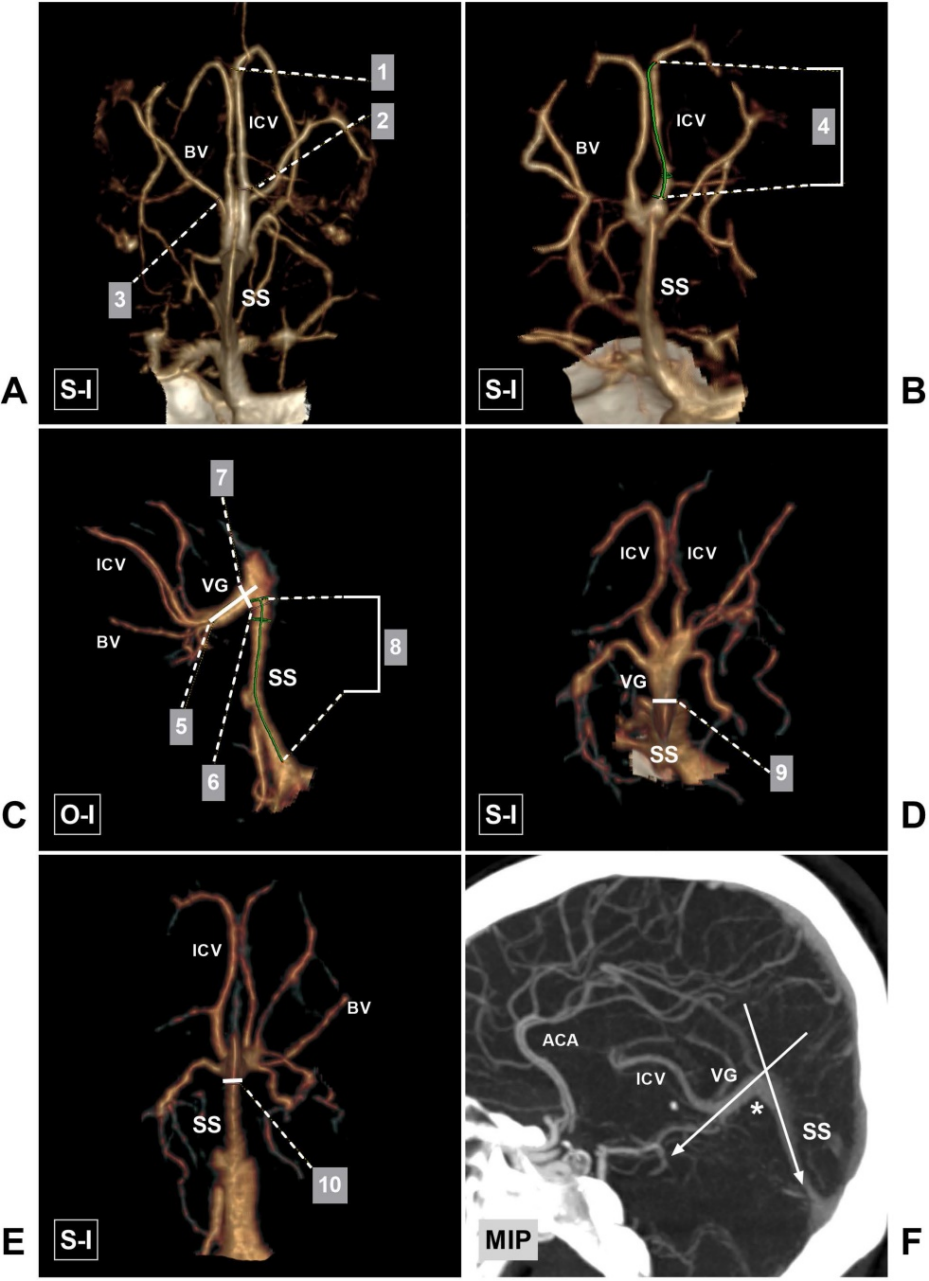
** Parameters measured in this study. A,** CTA shows the sites at which the diameter of the beginning (1) and termination (2) points of the ICV and the termination (3) point of the BV were measured. **B,** CTA shows the length (4) of the ICV. **C,** CTA shows the length (5) of the VG, the width (6) of the beginning of the SS, the thickness (7) of the VG termination point, and the length (8) of the SS. **D,** CTA shows the width of the VG (9). **E,** CTA shows the thickness of the beginning of the SS (10). F, CTA shows the angle at the VG and SS transition area (asterisk). **Abbreviations:** ACA, anterior cerebral artery; BV, basal vein; CTA, computed tomography angiography; ICV, internal cerebral vein; MIP, maximum intensity projection; O-I, outside to inside; S-I, superior to inferior; SS, straight sinus; VG, vein of Galen.

The measured parameters included the diameter of the ICV at its beginning and termination points, the diameter of the BV termination point, the length of the ICV, the length of the VG, the width and thickness at the VG termination point, the width and thickness at the beginning of the straight sinus (SS), the length of the SS, and the angle at the VG and SS transition area. The beginning of the ICV was defined as the venous angle that was U-shaped and near the junction of the thalamostriate vein (TSV) and ICV. The angle at the VG and SS transition area is shown in Figure 2. If a lateral direct vein (LDV) existed, its terminal diameter (at the entrance to the ICV) was also measured.

### Count parameters

**Figure 3:**
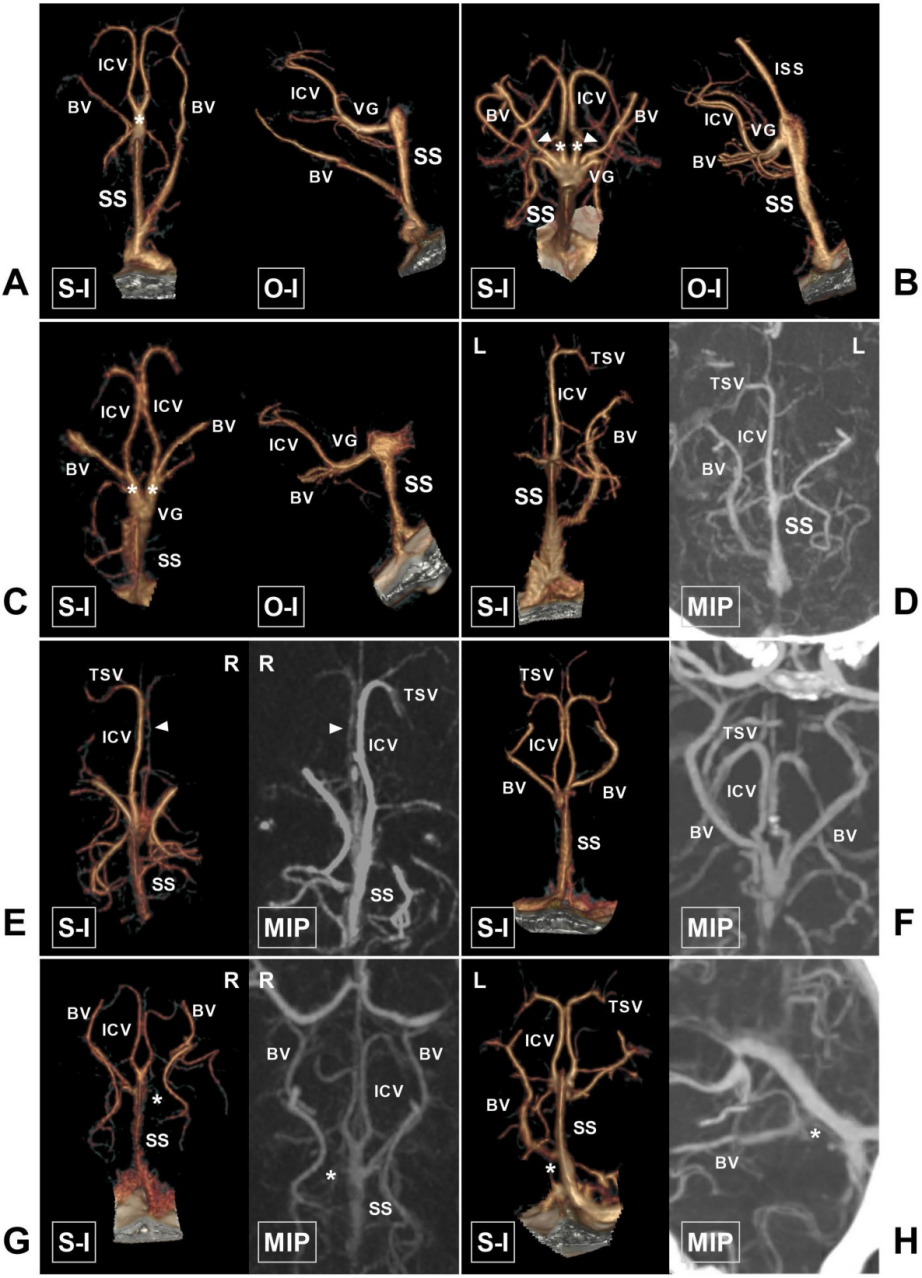
** The ICV and BV and their draining characteristics into the VG and SS. A,** CTA shows that the bilateral ICVs merge into one trunk (asterisk) before draining into the VG. **B,** CTA shows that the bilateral ICVs (asterisk) and BVs (arrowhead) drain into the VG. **C,** CTA shows that the ipsilateral ICV and BV merge into one trunk (asterisk) before draining into the VG. **D,** CTA and MIP show that the left ICV is absent. **E,** CTA and MIP show that the right ICV (arrowhead) is dysplastic. **F,** CTA and MIP show that the bilateral BVs are well developed. **G,** CTA and MIP show that the right BV is dysplastic (asterisk). **H,** CTA and MIP show that the left BV drains directly into the SS (asterisk). **Abbreviations:** BV, basal vein; CTA, computed tomography angiography; ICV, internal cerebral vein; ISS, inferior sagittal sinus; MIP, maximum intensity projection; O-I, outside to inside; S-I, superior to inferior; SS, straight sinus; TSV, thalamostriate vein; L, left; R, right; VG, vein of Galen.

**Figure 4:**
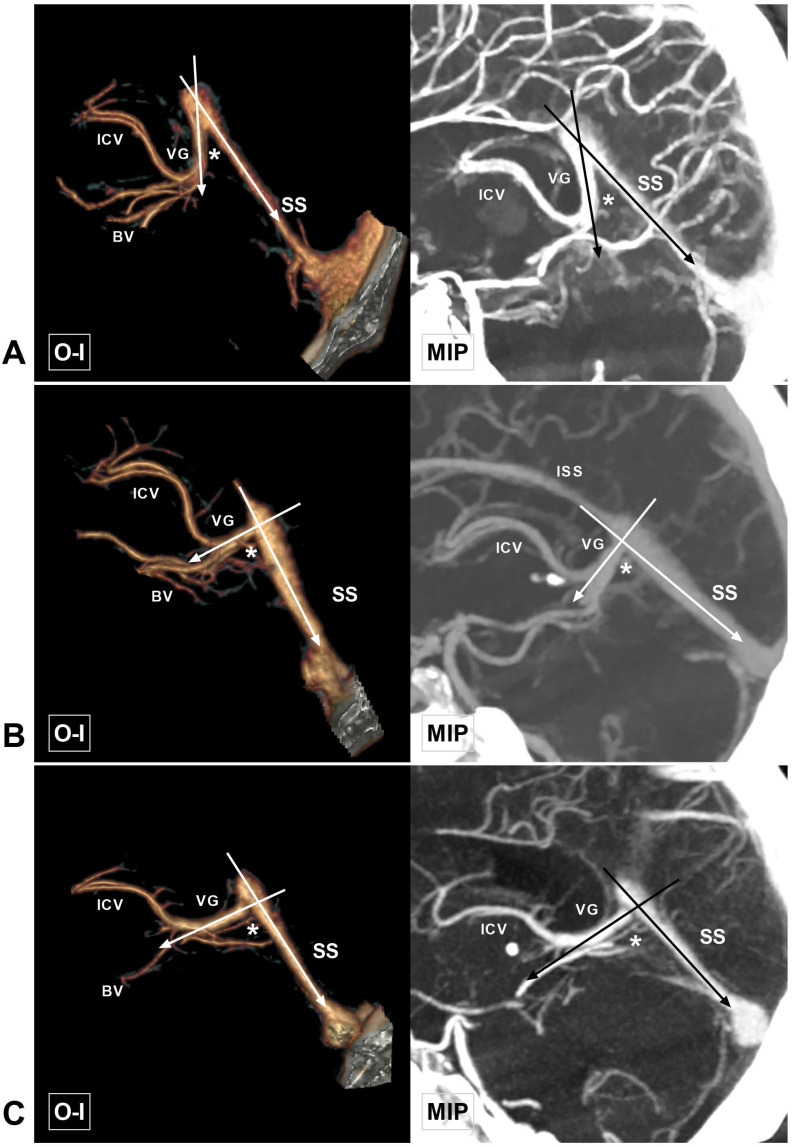
** Different angles at the VG and SS transition area. A,** CTA and MIP show an acute angle at the VG and SS transition area (asterisk). **B,** CTA and MIP show a right angle at the VG and SS transition area (asterisk). **C,** CTA and MIP show an obtuse angle at the VG and SS transition area (asterisk). **Abbreviations:** BV, basal vein; CTA, computed tomography angiography; ICV, internal cerebral vein; ISS, inferior sagittal sinus; MIP, maximum intensity projection; O-I, outside to inside; SS, straight sinus; VG, vein of Galen.

**Figure 5:**
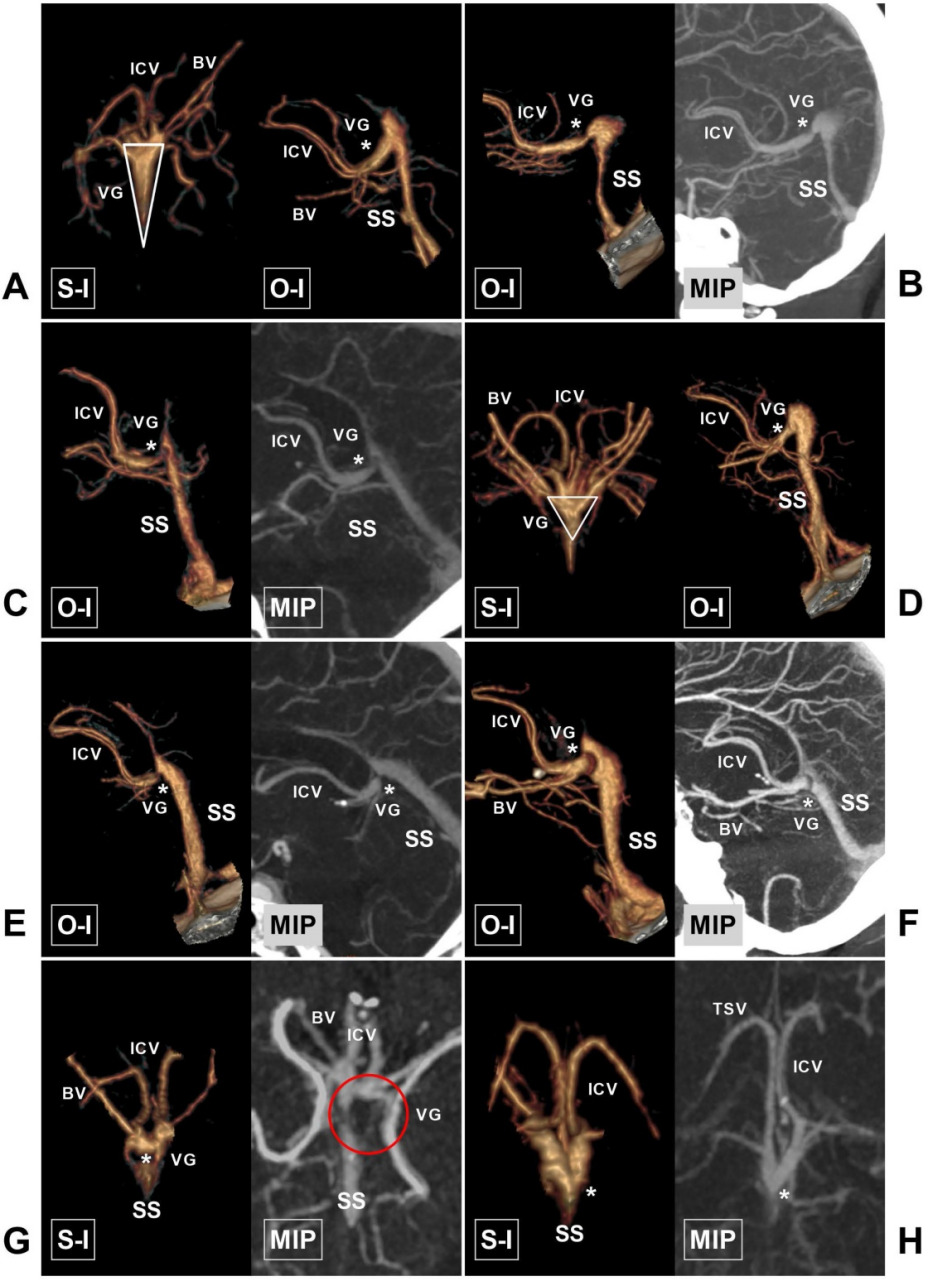
** Angioarchitecture of the VG. A,** CTA shows that the length of the VG (asterisk) is greater than the width, presenting an acute triangle. **B,** CTA and MIP show that the VG is stenotic (asterisk) at its SS transition area. **C,** CTA and MIP reveal an impression of the arachnoid granulation at the junction of the VG and SS. **D,** CTA shows that the cross sectional shape of the VG (asterisk) is an equilateral triangle. **E,** CTA and MIP show that the equilateral triangle VG is stenotic (asterisk) at its SS transition area. **F,** CTA and MIP reveal an impression of the arachnoid granulation (asterisk) at an equilateral angle VG. **G,** CTA and MIP show that the VG is irregular and drains to the SS through multiple tiny channels (asterisk and encircled area). **H,** CTA and MIP show that the VG and BV merge into a common trunk and drain directly to the SS (asterisk) in the absence of the VG. **Abbreviations:** BV, basal vein; CTA, computed tomography angiography; ICV, internal cerebral vein; MIP, maximum intensity projection; O-I, outside to inside; S-I, superior to inferior; SS, straight sinus; TSV, thalamostriate vein; VG, vein of Galen.

The observational parameters included the draining characteristics of the ICV and BV into the VG or SS (Figure 3), the angle at the VG and SS transition area (Figure 4), the angioarchitecture of the VG, and the origin of the LDV. The draining characteristics of the ICV and BV included the following: the ICV and BV separately emptied into the VG, the ICV and BV merged into one trunk and then drained into the VG, and the bilateral ICVs merged into one trunk and then drained into the VG. From the superior view, the cross sectional shape of the VG was classified into acute triangle, equilateral triangle, irregular, and absence of VG (Figure 5).

**Figure 6:**
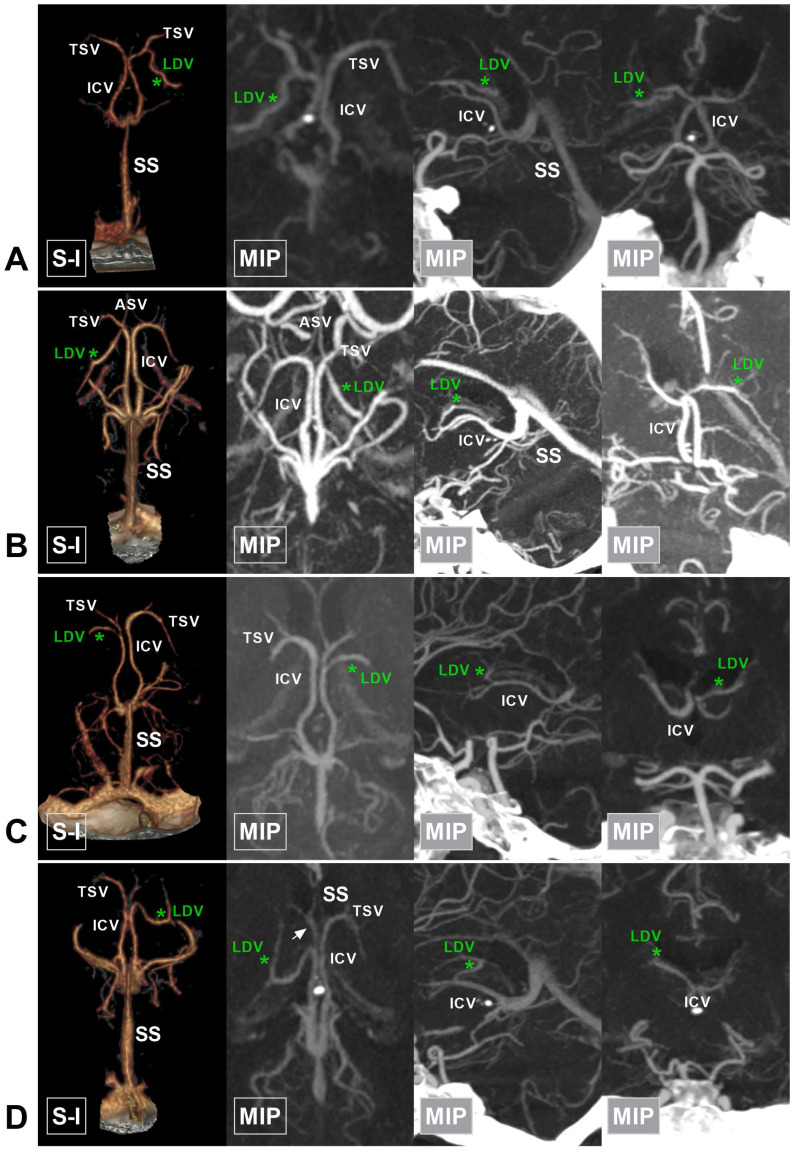
** The anterior type of LDV. A,** CTA and MIP show that the LDV (asterisk) drains into the TSV and courses posterolaterally. **B,** CTA and MIP show that the LDV (asterisk) drains into the confluence of the ASV and TSV. **C,** CTA and MIP show that the LDV (asterisk) drains into the ICV after the confluence of the ASV and TSV. **D,** CTA and MIP show that the LDV (asterisk) drains into the anterior half of the ICV (arrow). **Abbreviations:** ASV, anterior septal vein; CTA, computed tomography angiography; ICV, internal cerebral vein; LDV, lateral direct vein; MIP, maximum intensity projection; S-I, superior to inferior; SS, straight sinus; TSV, thalamostriate vein.

**Figure 7:**
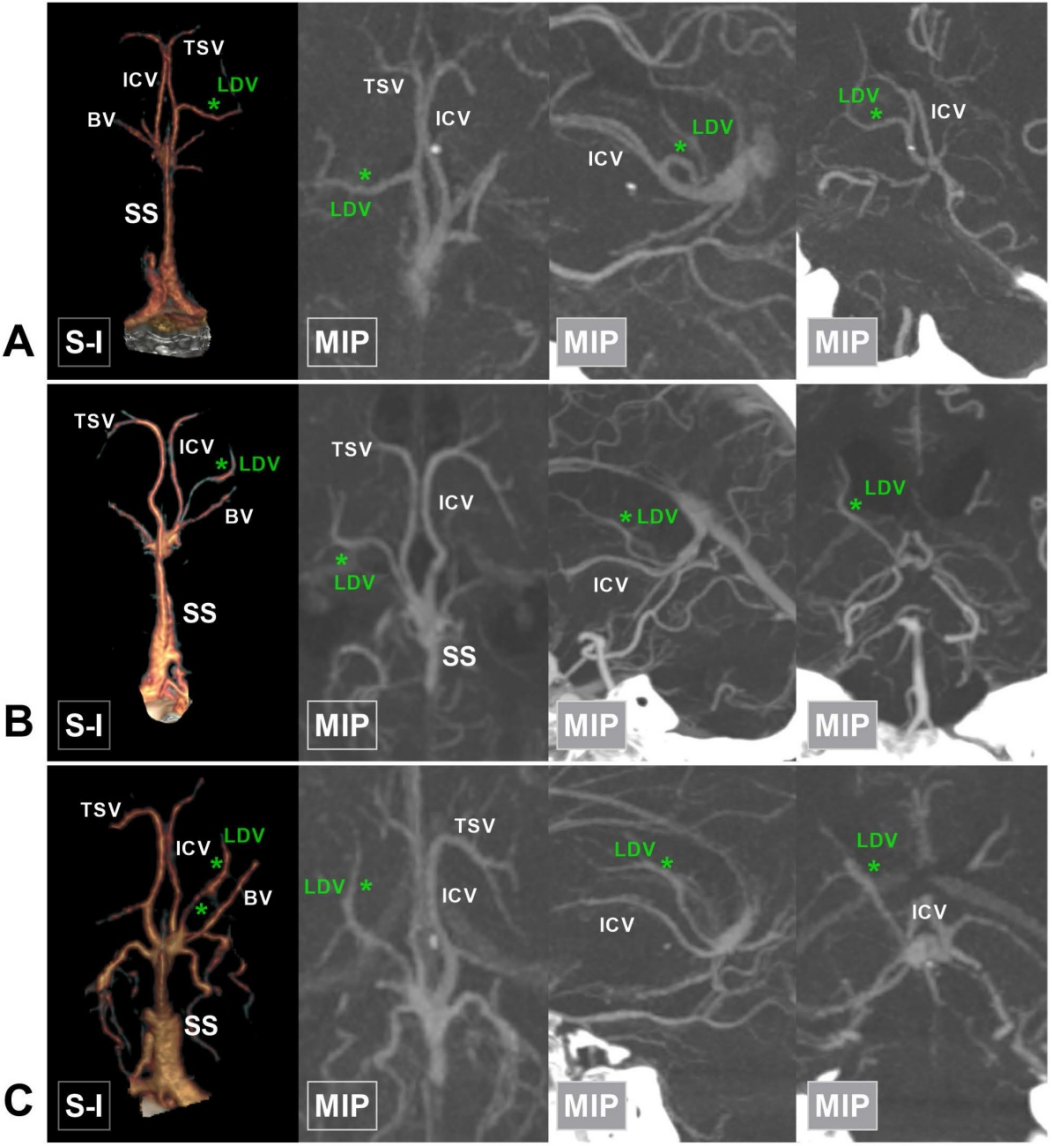
** The posterior type of LDV. A,** CTA and MIP show that the LDV (asterisk) drains into the middle of the ICV and extends laterally. **B,** CTA and MIP show that the LDV (asterisk) drains into the posterior half of the ICV. **C,** CTA and MIP show that the LDV (asterisk) drains into the confluence of the ICV and BV. **Abbreviations:** BV, basal vein; CTA, computed tomography angiography; ICV, internal cerebral vein; LDV, lateral direct vein; MIP, maximum intensity projection; SS, straight sinus; TSV, thalamostriate vein.

**Figure 8:**
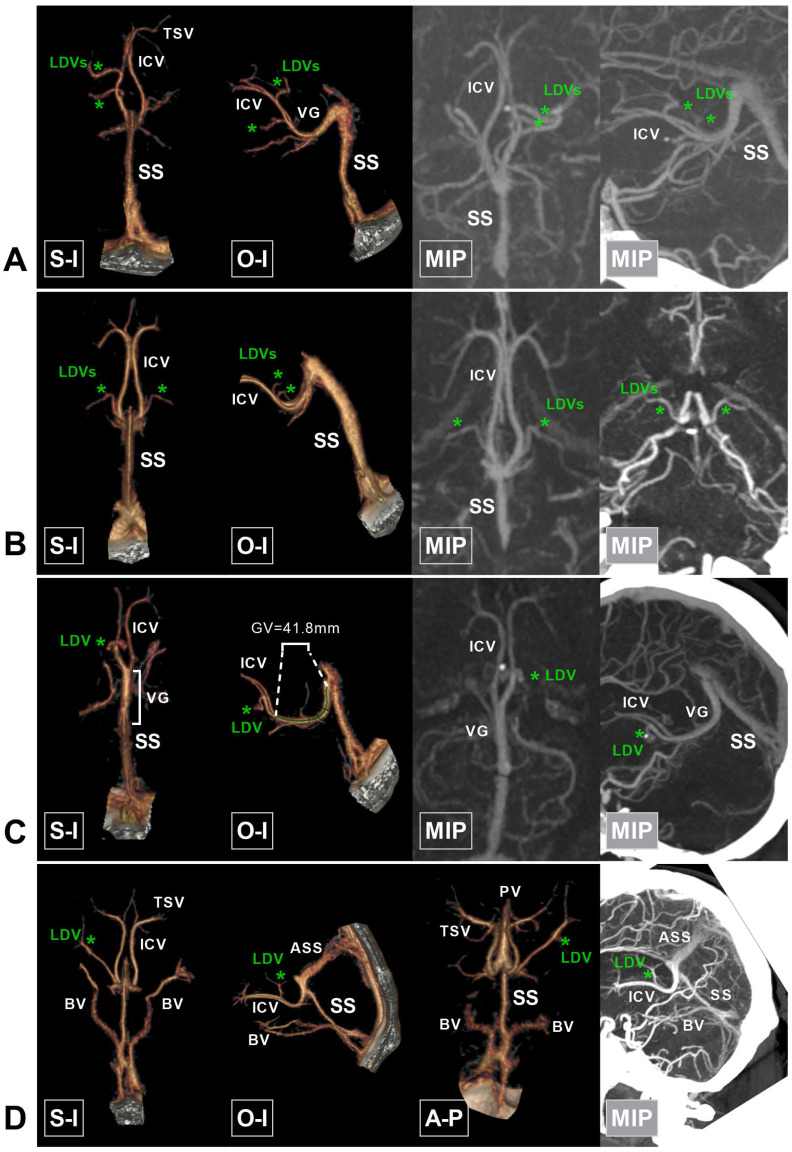
** Rare variations in the deep cerebral veins around the VG. A,** CTA and MIP show duplicate LDVs (asterisk) draining into the left ICV. **B,** CTA and MIP show that the LDVs (asterisk) drain into the terminal part of the bilateral ICVs. **C,** CTA and MIP show a very long VG (41.8 mm) and that the bilateral ICVs merge early. **D,** An accessory SS (ASS) is noted above the SS, which has the same draining function as the SS. The SS decreases accordingly. The bilateral BVs drain directly into the terminal portion of the SS. **Abbreviations:** A-P, anterior to posterior; ASS, accessory straight sinus; BV, basal vein; CTA, computed tomography angiography; ICV, internal cerebral vein; MIP, maximum intensity projection; O-I, outside to inside; S-I, superior to inferior; SS, straight sinus; TSV, thalamostriate vein; VG, vein of Galen.

In addition, stenosis or an impression of the arachnoid granulation (articular shape) at the junction of the VG and SS was also recorded. Stenosis of the VG and SS junction was defined as a width and thickness less than 2 mm. The LDV was classified into anterior and posterior types according to its relationship with the midpoint of the ICV (Figure 6-7). Other rare venous variations identified in this study were also recorded (Figure 8).

### Statistical analysis

Statistical assessment was performed using GraphPad Prism (8.4). Continuous variables are expressed as the mean ± standard deviation, and differences were assessed with a t test. P<0.01 was considered to indicate a statistical difference. The relationship between independent variables and dependent variables was analyzed using linear regression.

## Results

### General information

The patients were aged from 17 to 78 years (mean: 42.3 years). There were 46 females (46%, 46/100) and 54 males (54%, 54/100). There were 30 healthy cases (30%, 30/100), 17 cases of hemorrhagic diseases (17%, 26/100), and 53 cases of ischemic diseases (53%, 53/100).

### Measurement data

**Figure 9:**
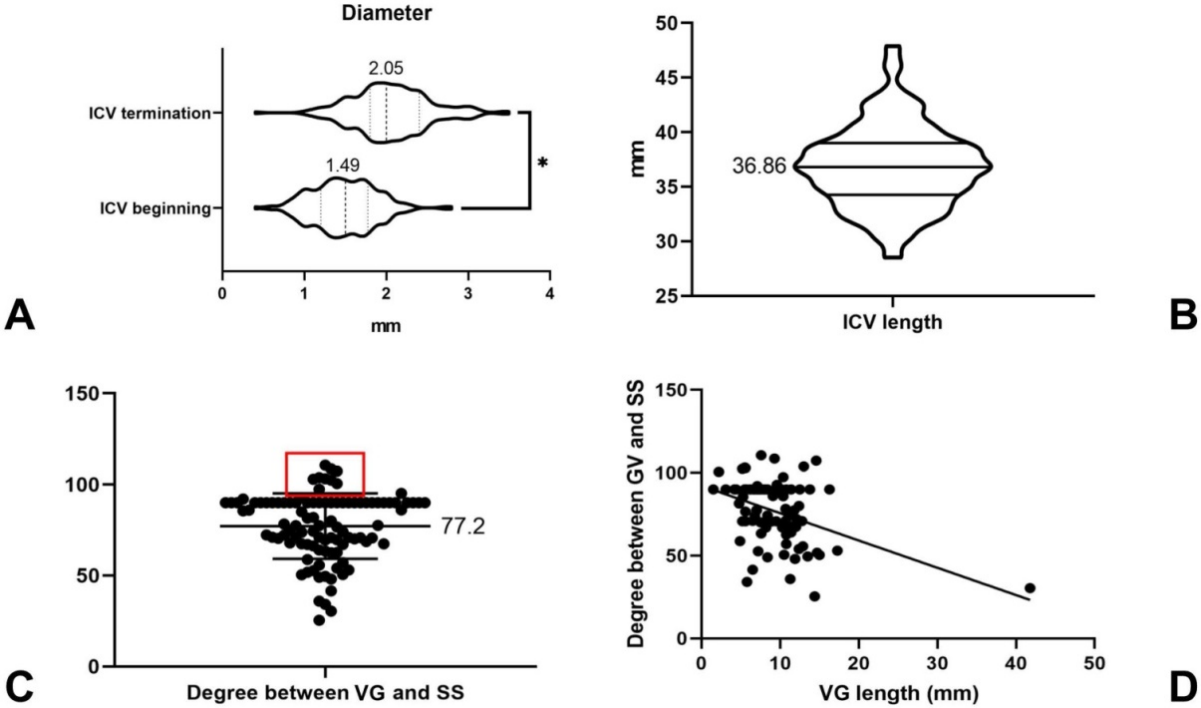
** Statistical results. A,** The box and violin plot generated by an unpaired t test, which shows that the ICV diameter at the beginning is less than its terminal diameter (P <0.01). **B,** The box and violin graph shows the length of the ICV, averaging 36.86 mm. **C,** The plot shows the scatter distribution of the angle at the VG and SS transition area. The red rectangular area denotes the distribution of obtuse angles. **D,** Linear regression analysis shows that Y = -1.648*X + 92.18; the deviation from zero is significant (P value<0.01). **Abbreviations:** ICV, internal cerebral vein; SS, straight sinus; VG, vein of Galen.

Of the 100 cases, thus in a total of 200 hemispheres, the diameter of the beginning of the ICV could be measured in 196 hemispheres (98%, 196/200), ranging from 0.4 to 2.8 mm (1.49 ± 0.39 mm). The diameter of the ICV termination point was measured in 198 hemispheres (98%, 198/200), ranging from 0.4 to 3.5 mm (2.05 ± 0.47 mm). There was statistical significance with regard to the diameter of the ICV at its beginning and termination points (P <0.01), indicating that the diameter of the ICV gradually increased (Figure 9A). The ICV length could be measured in 196 hemispheres, ranging from 28.5 to 47.9 mm (36.86 ± 3.74 mm) (Figure 9B).

Thirty-eight (19.0%) LDVs were identified in the 200 hemispheres, of which 16 were the posterior type (42.1%, 16/38) and 22 were the anterior type (57.9%, 22/38). The terminal diameter of the LDV ranged from 0.5 to 2.1 mm (1.20 ± 0.40 mm). The terminal diameter of the BV could be measured in 188 hemispheres, ranging from 0.5-4.3 mm (1.65 ± 0.61 mm).

The length of the VG could be measured in 88 (88%, 88/100) cases, ranging from 1.5 to 41.8 mm (9.30 ± 4.76 mm). The width and thickness of the VG termination point could be measured in 74 cases, with widths ranging from 0.8 to 6.8 mm (2.89 ± 1.07 mm) and thicknesses ranging from 1.4 to 8.8 mm (3.75 ± 1.56 mm). The thickness and width of the beginning of the SS ranged from 1.1 to 6.3 mm (3.08 ± 0.94 mm) and 1.2 to 10 mm (5.55 ± 1.88 mm), respectively. The length of the SS ranged from 30.2 to 57.8 mm (43.6 ± 6.37 mm). The angle at the VG and SS transition area ranged from 25.4 to 110.6° (77.2 ± 18.0°), among which 57% (57/100) were acute angles, 31% (31/100) were right angles, and 12% (12/100) were obtuse angles (Figure 9C). The measurement data are summarized in Table 1.

### Count data

Of the 200 cerebral hemispheres, ICV absence was identified in 2 (1%, 2/200) hemispheres, and the anterior portion of the ICV was absent in 2 (1%, 2/200) hemispheres. Dysplasia of the BV was identified in 12 (6%, 12/200) hemispheres. Stenosis or an impression of the arachnoid granulation of the VG termination point were identified in 16 (16%, 16/100) and 26 (26%, 26/100) hemispheres, respectively. Absence of the VG was identified in 6 cases (6%, 6/100). Irregular, elongated, acute triangle, and equilateral triangle VGs were identified in 6 (6%, 6/100), 1 (1%, 1/100), 38 (38%, 38/100), and 49 (49%, 49/100) cases, respectively.

The draining characteristics of the ICV and BV into the VG or SS could be identified in 190 (85%, 190/200) hemispheres. The ICV and BV emptied separately into the VG in 98 (49%, 98/200) hemispheres. The ICV and BV converged into one trunk and then drained into the VG in 51 hemispheres (25.5%, 51/200). The bilateral ICVs converged into one trunk and then drained into the VG in 34 hemispheres (17%, 34/200). The ICV drained into the VG and the BV drained into the SS in 7 hemispheres (3.5%, 7/200). An accessory SS was identified in 3 (3%, 3/100) cases.

### Linear regression analysis

The VG could be measured in 88 cases. The length of the VG and the angle at the VG and SS transition area were analyzed using linear regression. Linear regression results showed that the angle was negatively associated with the length of the VG (β = -1.648, p < 0.01). This indicates that with the extension of VG, the angle at the VG and SS transition area decreased (Figure 9D).

## Discussion

**Figure 10:**
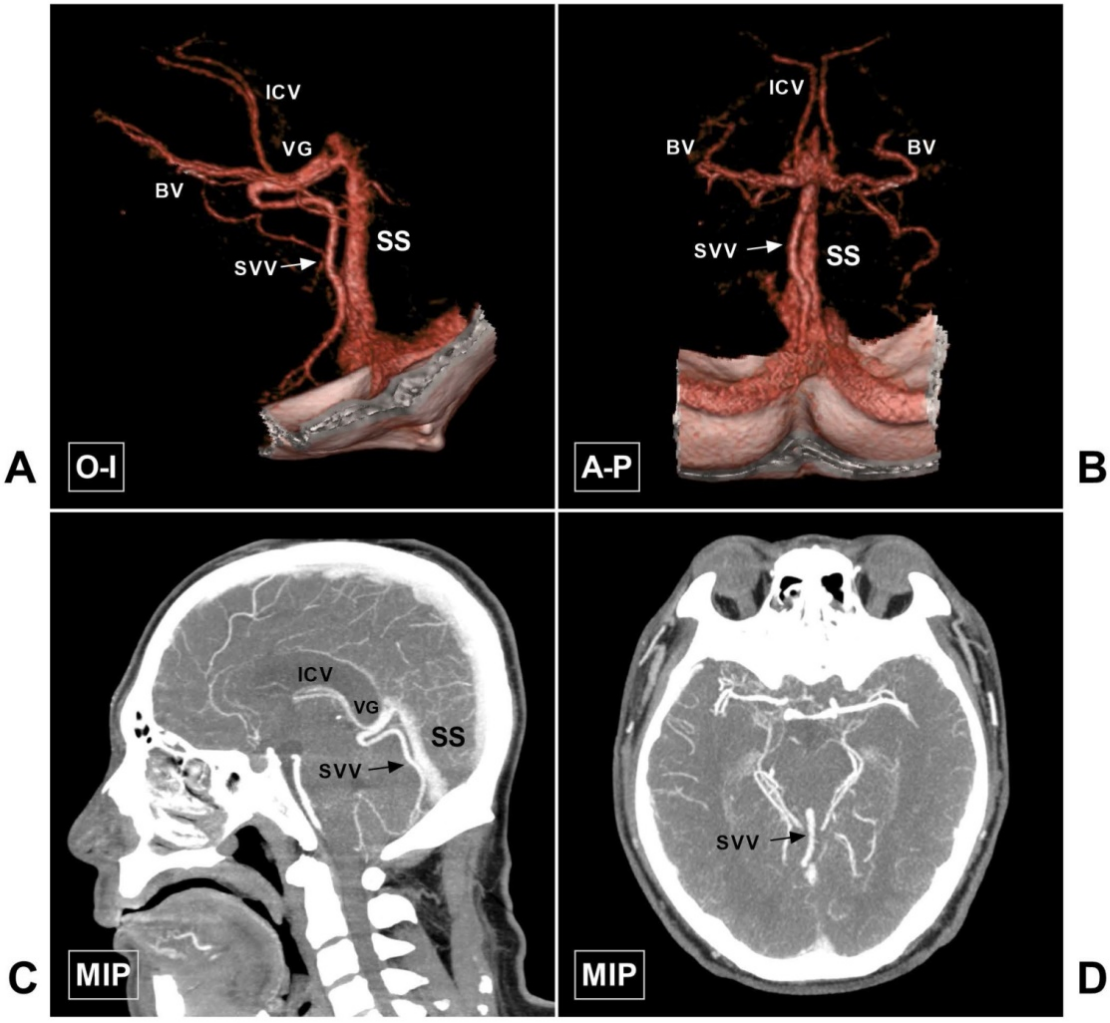
** Large SVV. A-B,** CTA in O-I and A-P view show a large SVV (arrow), which drains to the VG. **C-D,** MIP in sagittal and axial view also shows the large SVV (arrow). **Abbreviations:** A-P, anterior to posterior; BV, basal vein; CTA, computed tomography angiography; ICV, internal cerebral vein; MIP, maximum intensity projection; O-I, outside to inside; SS, straight sinus; SVV, superior vermin vein; VG, vein of Galen.

The CDV system mainly refers to the veins around the VG, including the ICV, BV, and VG, of which the VG is the core. The VG mainly receives blood flow from the bilateral ICVs and BVs and then empties into the SS. In addition, the VG and SS receive blood flow from the superior vermian vein, the superior cerebellar vein, the tectal and pineal veins, and the internal occipital vein 5. The CDV system is characterized by diverse anatomical variations. However, the ICV, BV, and VG are relatively constant. Some veins can be very slim or even absent, or they can be very large. An illustrative case of the large superior vermian vein is shown in Figure 10.

The ICV begins at the junction of the anterior septal vein (ASV) and the TSV, and no bridging veins connect the bilateral ICVs. Other major tributaries that join the ICV include the LDV and the medial atrial vein (MAV). The ICV can be dysplastic at the anterior portion or even absent. In our study, the absence of the ICV was identified in 2 hemispheres (1%, 2/200), and dysplasia of the anterior portion (concurrent with the LDV) was identified in 2 hemispheres (1%, 2/200).

Currently, there have been few anatomical studies of the ICV. According to a study by Chaynes et al. (2003), the diameter of the ICV increased as it continued posteriorly. The terminal diameter of the ICV ranged from 1.4 to 3.9 mm (mean: 2.67 mm) 3. However, the diameter of the ICV termination point in our study ranged from 0.4 to 3.5 mm (2.05 ± 0.47 mm), which was slightly smaller than that of Chaynes et al.'s study, possibly because our study was conducted in an Asian population.

The diameter of the beginning of the ICV at the venous angle was also measured in this study, ranging from 0.4 to 2.8 mm (1.49 ± 0.39 mm). There was statistical difference with regard to the diameter of the ICV at its beginning and termination points (P <0.01). The length of the ICV was also measured, ranging from 28.5 to 47.9 mm (36.86 ± 3.74 mm). These anatomical data greatly enriched our anatomical knowledge of the ICV.

Sometimes, the ICV gives rise to the LDV, which is also known as the thalamocaudate vein and is located on the floor of the body of the lateral ventricle and enters the ICV at various levels 6. In Brzegowy et al.'s (2019) study, the LDV was identified in 22% of the hemispheres evaluated and usually terminated at the middle third of the ICV (65.45%) 7. In our study, the incidence of an LDV was 19.0%, which was similar to that of Brzegowy et al.'s study 7. To prevent unintentional injury to the LDV during endoscopic surgery involving the lateral ventricle, different kinds of classifications of the LDV were proposed. As CTA is not good at displaying the anatomical relationship between the ventricular parenchyma and the LDV, this study only generally divided the LDV into anterior and posterior types.

In addition to the ICV, the BV is also a very important CDV. It typically lies on the surface of the base of the brain, goes around the brain stem, and then drains posteriorly into the VG. The BV is formed by longitudinal anastomoses of three primitive veins: the telencephalic, diencephalic, and mesencephalic veins 8. The BV is not as constant as the ICV. According to Watanabe et al.'s classification, the BV was classified into 3 types based on its anatomical variations 9. Anatomically, the BV is divided into three segments, 10 which were clearly displayed by CTA in this study. In this study, we measured the terminal diameter of the BV, determined whether the third segment of the BV existed, and evaluated its draining characteristics. In Chaynes et al.'s study (2003), BV was encountered in 74% of the cadavers evaluated. The diameter at its terminus ranged from 1.3 to 3.2 mm (mean: 2.21 mm) 3, which is wider than that of our study. This difference might be due to the method of measuring or the population difference. In addition, this study found that the third segment of the BV was dysplastic in 6% of the studied hemispheres, and the BV directly drained to the SS in 3.5% of the studied hemispheres.

The VG is the core of the CVD system and courses posteriorly and superiorly before emptying into the SS with the inferior sagittal sinus. In Chaynes et al.'s study, the length of the VG varied from 3.1 to 25 mm (mean: 10.33 mm) 3. In our study, the length of the VG ranged from 1.5 to 41.8 mm (9.30 ± 4.76 mm). Although the average length was similar to that of Chaynes' report, an extremely long case of VG (41.8 mm) was noted in our study (Figure 8C), indicating the enormous variation in the VG.

The morphology of the VG has never been studied before. In the superior view, we found that the VG was triangular in most of the cases (87%). In the case of a long VG, the cross sectional morphology was an acute triangle. With decreasing length, it became an equilateral triangle and ultimately developed an irregular shape or even disappeared. There has been no specific previous research on the junction of the VG and SS. This study found that the junction of the VG and SS was wide and unobstructed in only 58% of the cases. Stenosis or an impression of the arachnoid granulation of the junction of the VG and SS was identified in 42% of the cases. In this study, the threshold of VG and SS junction stenosis was defined as 2 mm. Preoperative identification of stenosis or an impression of the arachnoid granulation of the VG and SS junction has important clinical implications. For patients with stenosis or an impression of the arachnoid granulation of the VG and SS junction, transvenous management of the vascular lesion in this region would be quite challenging 11.

The draining characteristics of the BV and ICV into the VG vary greatly in different patients. Usually, the BV drains into the VG more inferiorly than the ICV does 3. In this study, the ICV and BV drained separately into the VG in 49% of the cases. The ICV and BV merged into a common trunk and then drained into the VG in 25.5% of the cases. In addition, the bilateral ICVs converged and then drained into the VG in 17% of the cases.

The SS is the continuation of the VG. The length and morphology of the SS are not constant. According to Saxena et al.'s study, the average length was 50 mm (40-69 mm) 12. The length of the SS in our study ranged from 30.2 to 57.8 mm (43.6 ± 6.37 mm), which is also shorter than that in Saxena et al.'s study. Moreover, we found that the SS was flat, with its thickness and width at its beginning ranging from 1.1 to 6.3 mm (3.08 ± 0.94 mm) and 1.2 to 10 (5.55 ± 1.88 mm), respectively. In addition, there are some other variations. For instance, there could be double SSs 13. An accessory SS with the same function as an SS is also a rare variation (Figure 8D).

This study also investigated the angle at the VG and SS transition area. In Chaynes et al.'s study, the angle varied from 16° to 117° (mean: 75.2°), and obtuse angles were rare 3. Our study findings are also consistent with the results of their research. Moreover, linear regression analysis showed that the angle negatively correlated with the length of the VG. Notably, it would be meaningful to further study the angle at the VG and SS transition area, which, in combination with the diameter of the VG and SS junction, could assist in predicting the degree of patency in deep venous drainage.

## Figures and Tables

**Table 1:** Summary of count data

Parameters	Measured value
Diameter of the beginning of the ICV	0.4-2.8 mm (1.49 ± 0.39 mm)
Diameter of the termination of the ICV	0.4-3.5 mm (2.05 ± 0.47 mm)
ICV length	28.5-47.9 mm (36.86 ± 3.74 mm)
Diameter of the termination of the LDV	0.5-2.1 mm (1.20 ± 0.40 mm)
Diameter of the termination of the BV	0.5-4.3 mm (1.65 ± 0.61 mm)
VG length	1.5-41.8 mm (9.30 ± 4.76 mm)
Width of the termination of the VG	0.8-6.8 mm (2.89 ± 1.07 mm)
Thickness of the termination of the VG	1.4-8.8 mm (3.75 ± 1.56 mm)
Width of the beginning of the SS	1.2-10 mm (5.55 ± 1.88 mm)
Thickness of the beginning of the SS	1.1-6.3 mm (3.08 ± 0.94 mm)
SS length	30.2-57.8 mm (43.6 ± 6.37 mm)
Angle at the VG and SS transition area	25.4-110.6° (77.2 ± 18.0°)

**Abbreviations:** BV, basal vein; ICV, internal cerebral vein; LDV, lateral direct vein; SS, straight sinus; VG, vein of Galen.
